# Right ventricular function and vasoactive peptides for early prediction of bronchopulmonary dysplasia

**DOI:** 10.1371/journal.pone.0257571

**Published:** 2021-09-22

**Authors:** Roland P. Neumann, Sven M. Schulzke, Christian Pohl, Sven Wellmann, Boris Metze, Ann-Katrin Burdensky, Vinzenz Boos, Payman Barikbin, Christoph Bührer, Christoph Czernik

**Affiliations:** 1 Department of Neonatology, University Children’s Hospital Basel UKBB, University of Basel, Basel, Switzerland; 2 Department of Neonatology, University Regensburg Children’s Hospital (KUNO), University of Regensburg, Regensburg, Germany; 3 Department of Neonatology, Charité - Universitätsmedizin Berlin, Berlin, Germany; 4 Department of Neonatology, Hospital Zollikerberg, Zollikerberg, Switzerland; 5 Department of Pediatrics, Vivantes Hospital Friedrichshain, Berlin, Germany; Hopital Robert Debre, FRANCE

## Abstract

**Background:**

To assess the prognostic value of early echocardiographic indices of right ventricular function and vasoactive peptides for prediction of bronchopulmonary dysplasia (BPD) or death in very preterm infants.

**Methods:**

Prospective study involving 294 very preterm infants (median [IQR] gestational age 28.4 [26.4–30.4] weeks, birth weight 1065 [800–1380] g), of whom 57 developed BPD (oxygen supplementation at 36 weeks postmenstrual age) and 10 died. Tricuspid annular plane systolic excursion (TAPSE), right ventricular index of myocardial performance (RIMP), plasma concentrations of mid-regional pro-atrial natriuretic peptide (MR-proANP) and C-terminal pro-endothelin-1 (CT-proET1) were measured on day 7 of life.

**Results:**

RIMP was significantly increased (median [IQR] 0.3 [0.23–0.38] vs 0.22 [0.15–0.29]), TAPSE decreased (median [IQR] 5.0 [5.0–6.0] vs 6.0 [5.4–7.0] mm), MR-proANP increased (median [IQR] 784 [540–936] vs 353 [247–625] pmol/L), and CT-proET1 increased (median [IQR] 249 [190–345] vs 199 [158–284] pmol/L) in infants who developed BPD or died, as compared to controls. All variables showed significant but weak correlations with each other (*r*_*S*_ -0.182 to 0.359) and predicted BPD/death with similar accuracy (areas under receiver operator characteristic curves 0.62 to 0.77). Multiple regression revealed only RIMP and birth weight as independent predictors of BPD or death.

**Conclusions:**

Vasoactive peptide concentrations and echocardiographic assessment employing standardized measures, notably RIMP, on day 7 of life are useful to identify preterm infants at increased risk for BPD or death.

## Introduction

Despite major advances in perinatal care, the prevalence of bronchopulmonary dysplasia (BPD) remains high, presumably due to the improved survival of extremely low gestational age infants [1–3]. BPD is associated with long-term respiratory morbidity, reflected by prolonged need of supplemental oxygen, recurrent respiratory infections, reduced exercise capacity, and increased risk of asthma-like symptoms in childhood [4–6]. Early lung injury disrupts normal alveolarization leading to simplification of the distal lung airspaces with enlarged alveoli and a variable degree of alveolar septal fibrosis [7]. Furthermore, pulmonary vasculature undergoes structural remodelling including medial hypertrophy and rarefication of vessels with abnormal branching and distribution within the interstitium [7,8].

Early prognostication of the BPD risk is desirable in order to adapt clinical management and to identify high-risk infants potentially eligible for inclusion in early-phase therapeutic trials. However, prediction of BPD development is difficult due to the complex pathophysiology of the disease including genetic and perinatal risk factors.

Pulmonary hypertension complicates BPD and contributes significantly to increased morbidity and mortality rates in infants with BPD [9]. Thus, echocardiographic evaluation of infants dependent on oxygen at 36 weeks is common practice in many neonatal units [10]. Moreover, it has recently been shown that elevated pulmonary pressures in the first weeks of life are associated with subsequent BPD development [9,11,12]. However, identification of infants with elevated pulmonary pressures at an early age remains difficult. Typical echocardiographic findings such as 1) right ventricular hypertrophy, 2) flattening of interventricular septum, 3) presence of tricuspid regurgitation in the absence of pulmonary stenosis, and 4) elevated right ventricular pressure as estimated by Doppler studies of tricuspid regurgitation jet are only inconsistently present.

Recognizing the important role of early pulmonary vascular disease in BPD pathophysiology, we aimed to explore the role of vasoactive peptides implicated in pulmonary hypertension and echocardiographic markers of right ventricular (RV) function in preterm infants. In recent studies, right ventricular index of myocardial performance (RIMP) as well as tricuspid annular plane systolic excursion (TAPSE) appeared to be promising echocardiographic indices for identification of infants with subsequent BPD development [11,13]. The vasoactive peptides endothelin-1 and natriuretic peptide are associated with pulmonary hypertension in adults [14]. They have previously been also studied in preterm infants and are considered candidate biomarkers for early determination of the BPD risk [15–18]. However, both echocardiographic indices and vasoactive peptides have not yet been investigated simultaneously in the first week of life in preterm infants so it is unknown which single marker or combination of markers is the best to predict BPD.

In the current study, we hypothesized that the echocardiographic indices RIMP and TAPSE and the plasma biomarkers mid-regional pro-atrial natriuretic peptide (MR-proANP) and C-terminal pro-endothelin-1 (CT-proET1) measured on day 7 of life predict subsequent respiratory morbidity in preterm infants. The aims of the present study were 1) to investigate the ability of the above-mentioned echocardiographic indices and biomarkers to predict BPD at 36 weeks postmenstrual age (PMA) or death and 2) to test their association with prolonged respiratory morbidity i.e. duration of supplemental oxygen and duration of respiratory support.

## Methods

### Study population

This prospective observational study was conducted in two tertiary care perinatal centers at University Children’s Hospital Basel, Switzerland and Charité Universitätsmedizin Berlin, Germany. Preterm infants with a gestational age (GA) < 32 weeks were considered eligible for the trial. Exclusion criteria were congenital heart disease, congenital diaphragmatic hernia, esophageal atresia, or infants who died during the first week of life. All infants received intensive care based on local standard operating procedures and were examined without sedation in a supine position.

The study protocol was approved by both local institutional review boards (Ethikkommission der Charité, # EA2/072/08 and Ethics Committee of Northwestern and Central Switzerland, # EK233/13) and written informed parental consent was obtained before enrollment.

Perinatal variables and patients’ characteristics were collected from each infant’s chart, including BPD defined as a requirement for supplemental oxygen to maintain preductal arterial oxygen saturations of 92% at 36 + 0 weeks PMA [19,20]. Primary outcome of the study was the composite of BPD or death prior 36 weeks PMA (BPD/death).

### Echocardiographic measurements

Echocardiography was performed in Berlin using an Epiq 7G system with an S8-3 probe (Philips Medical Systems, Eindhoven, The Netherlands), or a Vivid S6 system with a 6S probe (GE Vingmed, Horten, Norway), and in Basel using a Mindray ZONARE Z.One PRO ultrasound machine with a 3–10 MHz transducer (Zonare Medical Systems, Mountain View, CA, USA). All scans were performed by experienced operators (RPN, CC, VB, PB).

We performed transthoracic echocardiography on day of life (DOL) 7 (± 2) days. The presence or absence of a patent ductus arteriosus (PDA) was determined by direct ductal imaging in the high left parasternal view with color Doppler mapping. TAPSE and RIMP were used to assess RV function. RIMP was measured by conventional pulsed Doppler using the method proposed by Tei et al. [21]. Therefore, pulsed-Doppler waveforms of tricuspid inflow were recorded from the parasternal four-chamber view and the “a” interval was measured between cessation and onset of the tricuspid inflow, while the right ventricular outflow patterns by pulsed-Doppler were visualized from the parasternal short-axis view in accordance with the recommendations of the American Society of Echocardiography [22]. The “b” interval was measured between onset and cessation of the RV outflow and RIMP was calculated as (a—b)/b. To minimize variations in heart rate, mean values were obtained by averaging a minimum of three consecutive cardiac cycles. The Doppler methodology as well as the feasibility and reproducibility of the RIMP measurements data are described in our recently published study [11].

### Biomarker analysis

Blood samples of 500 μL were drawn on DOL 7 (± 2 days) in EDTA tubes simultaneously with routine blood sampling. The samples were immediately transferred to the central laboratory of both study sites for centrifugation and storage at -80°C. Analyses for levels of MR-proANP and CT-proET1 were performed in batches using an automated immunofluorescent assay (BRAHMS KRYPTOR, Thermo Scientific, Henningsdorf, Germany) [23,24].

### Sample size estimation

Aiming for 85% power at the 5% significance level, we calculated a minimum required sample size of n = 292 using a multivariable linear regression model with duration of supplemental oxygen as the independent variable and the RIMP as the dependent variable, adjusting for demographic parameters birth weight, sex, growth retardation, and sepsis.

### Statistical analysis

SPSS software version 25.0 (IBM Corporation, Armonk, NY, USA) was used for statistical analysis. Patient characteristics and all measured data are reported as medians and interquartile ranges (IQR) or n (%) and compared using the Mann-Whitney U-test, Kruskal-Wallis test or Chi^2^-test or Fisher’s exact test as appropriate. Spearman rank correlation coefficients *r*_*S*_ were calculated to investigate the relationship between echocardiography parameters and vasoactive peptide concentrations. We performed logistic regression analysis with stepwise backward elimination of non-significant variables. The predictive values of RIMP, TAPSE and vasoactive peptides were estimated by the areas under receiver operating characteristic (ROC) curves. We investigated the association of echocardiographic indices and vasoactive peptides with duration of supplemental oxygen by univariable and multivariable linear regression analysis with stepwise backward elimination of non-significant variables. A *P*-value of < 0.05 was considered to be statistically significant.

## Results

### Subjects

**Fig 1:**
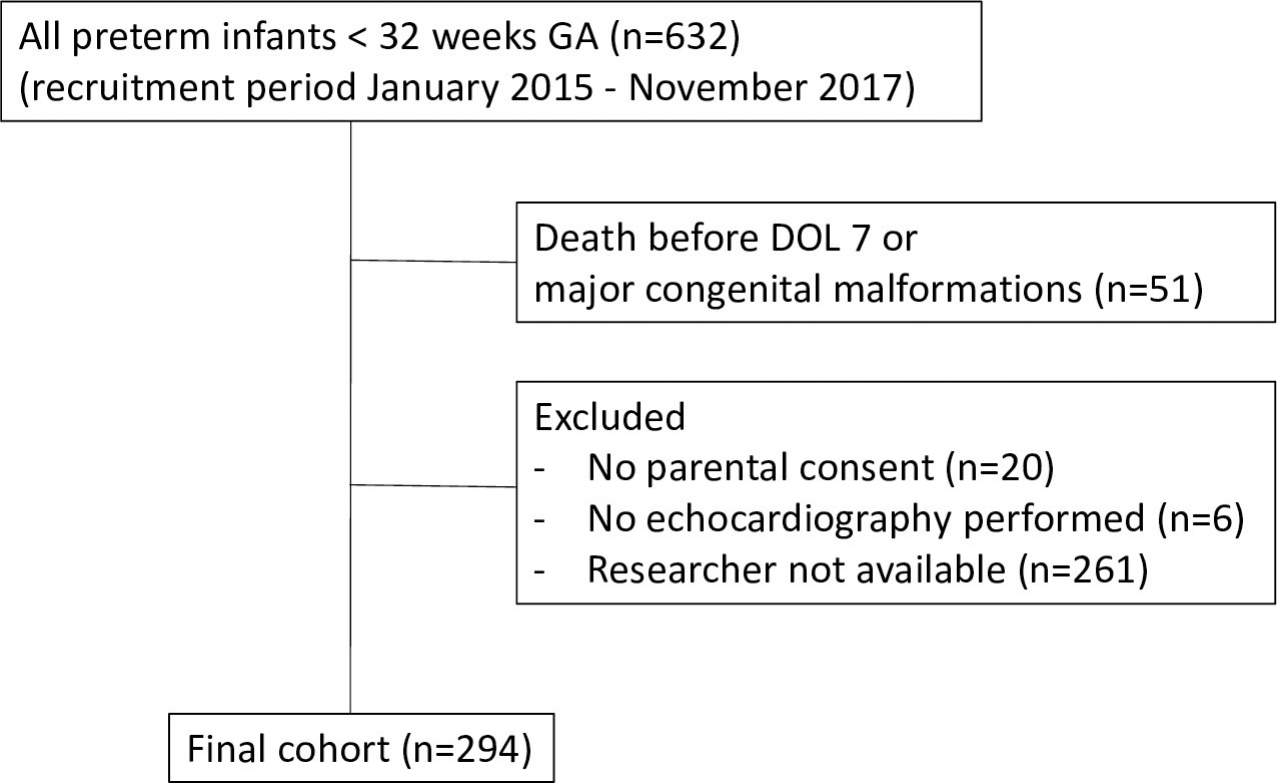
Recruitment flow chart.

A total of 294 preterm patients (90 from Basel, 204 from Berlin) were included into our study during January 2015 and November 2017. The recruitment flow chart can be found in Fig 1. The patient characteristics of included infants investigated by conventional echocardiography and their vasoactive peptide levels are shown in Table 1.

**Table 1:** Characteristics of the study population and comparison to infants with BPD/death and those without BPD.

	Study population n = 294	BPD/death n = 65	No BPD n = 229	*P*
Gestational age, weeks	28.4 (26.4–30.4)	25.1 (24.1–26.2)	29.5 (27.8–30.7)	< 0.01
Birth weight, grams	1065 (800–1380)	668 (570–780)	1170 (958–1439)	< 0.01
IUGR	67 (23%)	32 (49%)	35 (15%)	< 0.01
Male	156 (53%)	42 (60%)	114 (50%)	0.04
Fetal lung maturation	218 (74%)	41 (63%)	177 (77%)	0.015
Surfactant administration	180 (61%)	62 (95%)	118 (52%)	< 0.01
hsPDA	55 (19%)	34 (52%)	21 (9%)	< 0.01
Sepsis	66 (23%)	33 (52%)	33 (15%)	< 0.01
Duration of invasive mechanical ventilation, days	1 (0–4)	23 (8–37)	0 (0–2)	< 0.01
Duration of CPAP, days	20 (6–48)	52 (39–56)	16 (6–41)	0.015
Duration of supplemental oxygen, days	5 (1–29)	45 (27–61)	2 (1–17)	< 0.01
Duration of hospital stay, days	58 (43–80)	97 (76–118)	53 (41–66)	< 0.01

Data presented as median (IQR) or n (%). IUGR, intrauterine growth restriction defined as birth weight < 10^th^ percentile; hsPDA, hemodynamic significant patent ductus arteriosus; CPAP, continuous positive airway pressure.

About half of enrolled patients were male or required mechanical ventilation and 22% developed BPD. None of the infants received sedative, vasoactive or inotropic drugs when measurements were performed on DOL 7. Treatment for hemodynamically significant PDA with Indomethacin or Ibuprofen was performed in 55 (19%) infants at a median (IQR) age of 10 (7–16) days. BPD/death infants had a significantly lower GA and birth weight (*P* < 0.01). The rate of fetal lung maturation by antenatal corticosteroid administration (considered complete if the mother had received two injections of 12 mg betamethasone 24 hours apart and more than 24 hours before delivery) was higher in non-BPD infants (*P* < 0.01). Preterm infants with BPD/death received significantly longer mechanical ventilation and supplemental oxygen, and stayed longer in hospital (*P* < 0.01 for each factor). Ten infants died, seven of these had a fulminant sepsis, two patients died secondary to abdominal complications and one patient died from respiratory failure.

### Echocardiographic parameters and vasoactive peptides

**Fig 2:**
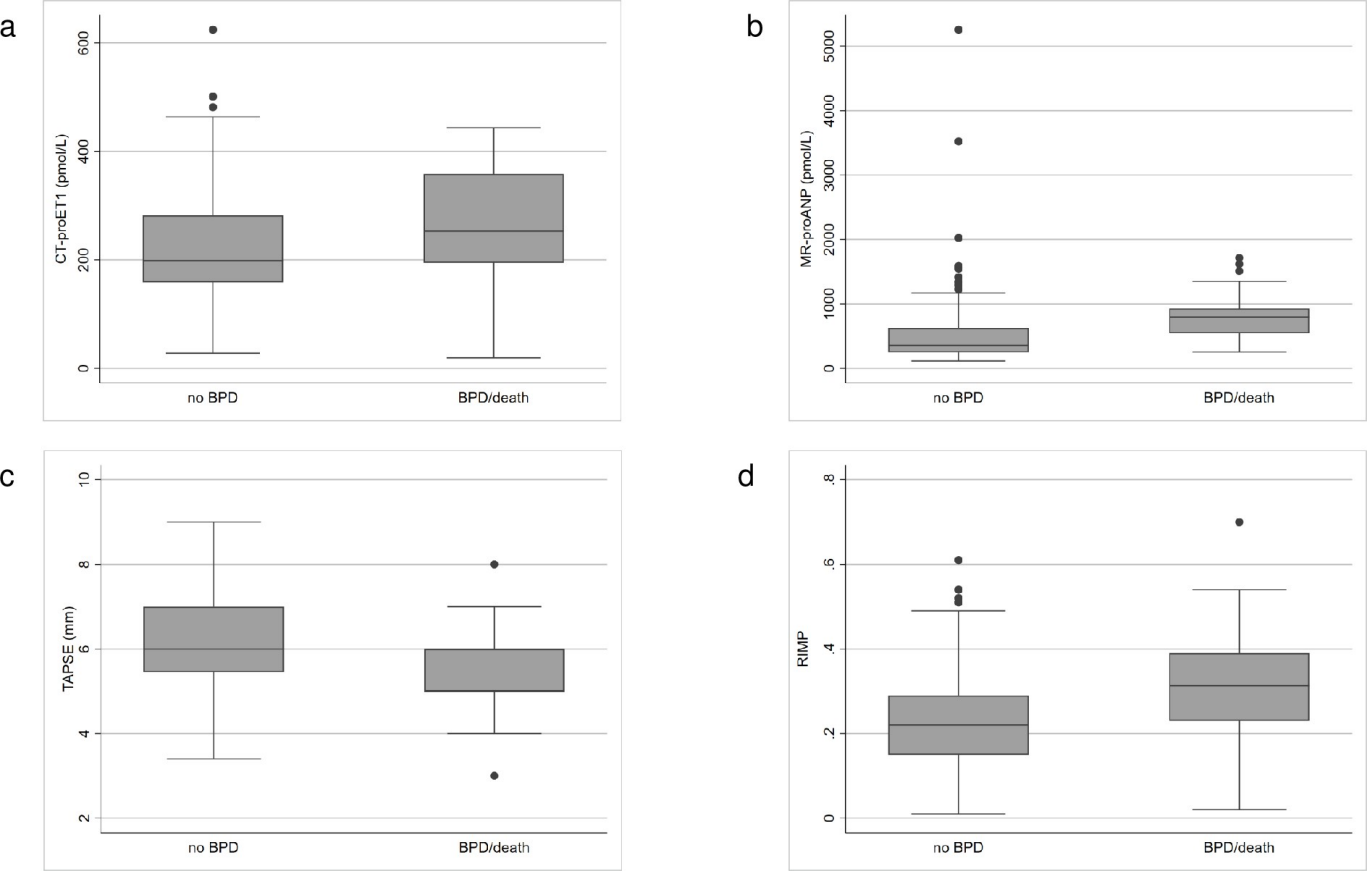
Echocardiographic parameters and vasoactive peptides in infants without BPD and with BPD/death. (a) CT-proET1 levels; (b) MR-proANP levels; (c) tricuspid annular plane systolic excursion (TAPSE); (d) right ventricular index of myocardial performance (RIMP).

Comparison of echocardiographic parameters and vasoactive peptides on DOL 7 between infants with BPD/death and without BPD are shown in Table 2 and Fig 2. RIMP values were higher and TAPSE values were lower in infants with BPD/death than in infants without BPD (*P* < 0.001). Both vasoactive peptides (MR-proANP and CT-proET1) were significantly higher in BPD/death infants as compared to controls (*P* = 0.016 and *P* < 0.001, respectively).

**Table 2:** Comparison of echocardiographic parameters and vasoactive peptides between infants with BPD/death and without BPD.

Parameter	BPD/death	No BPD	*P*
*Echocardiographic parameters*			
RIMP	0.3 (0.23–0.38)	0.22 (0.15–0.29)	<0.001
TAPSE, mm	5.0 (5.0–6.0)	6.0 (5.4–7.0)	<0.001
*Vasoactive peptides*			
MR-proANP, pmol/L	784 (540–936)	353 (247–625)	<0.001
CT-proET1, pmol/L	249 (190–345)	199 (158–284)	0.027

Data presented as median (IQR).

Additionally, we examined the correlation (*r*_*s*_) of RIMP with TAPSE, MR-proANP and CT-proET1. Vasoactive peptides were positively correlated with RIMP (*P*  < 0.001) and negatively correlated with TAPSE (*P* = 0.003), but all observed correlations were weak (TAPSE: *r*_*S*_ = –0.182; MR-proANP: *r*_*S*_ = 0.346; CT-proET1: *r*_*S*_ = 0.323) (Table 3).

**Table 3:** Spearman correlation coefficients (*r*_*S*_) between echocardiographic measurements and vasoactive peptide serum concentrations.

	RIMP	TAPSE	MR-proANP	CT-proET1
RIMP	1	-0.182 *P* < 0.01	0.346 *P* < 0.01	0.323 *P* < 0.01
TAPSE	-0.182 *P* < 0.01	1	-0.253 *P* < 0.01	-0.302 *P* < 0.01
MR-proANP	0.346 *P* < 0.01	-0.253 *P* < 0.01	1	0.359 *P* < 0.01
CT-proET1	0.323 *P* < 0.01	-0.302 *P* < 0.01	0.359 *P* < 0.01	1

### Impact of RV function and vasoactive peptides among patient groups

In a logistic regression model with birth weight, RIMP, TAPSE, MR-proANP and CT-proET1 in the equation, only birth weight and RIMP were independent predictors of BPD/death (*P* < 0.001 and *P* = 0.012). Lower birthweight and higher RIMP values predicted BPD/death. The likelihood of developing BPD/death can be calculated using both parameters in the equation specified by the estimates of regression coefficients in Table 4.


$$\text{P}\left(\text{BPD}/\text{death}\right)=\frac{1}{1+e^{-(}{}^{3.9-0.007*birthweight+4.04*RIMP)}}$$

**Table 4:** Impact of birth weight, RV function parameters and vasoactive peptides on logistic regression to estimate risk for BPD/death.

	ß	SE	*P*	Exp(ß)	95% CI for Exp(ß)	
					Lower	Upper
Birth weight	-0.008	0.001	0.000	0.992	0.990	0.995
RIMP	4.112	1.637	0.012	61.052	2.469	1509.389
TAPSE (mm)	0.113	0.236	0.634	1.119	0.704	1.778
MR-proANP	0.000	0.000	0.298	1.000	0.999	1.000
CT-proET1	-0.001	0.002	0.688	0.999	0.994	1.004
constant	4.177	1.699	0.014	65.171		

ß, estimated regression coefficient; SE, standard error; Exp(ß), exponentiation of estimated regression coefficient ß; CI, confidence interval.

**Fig 3:**
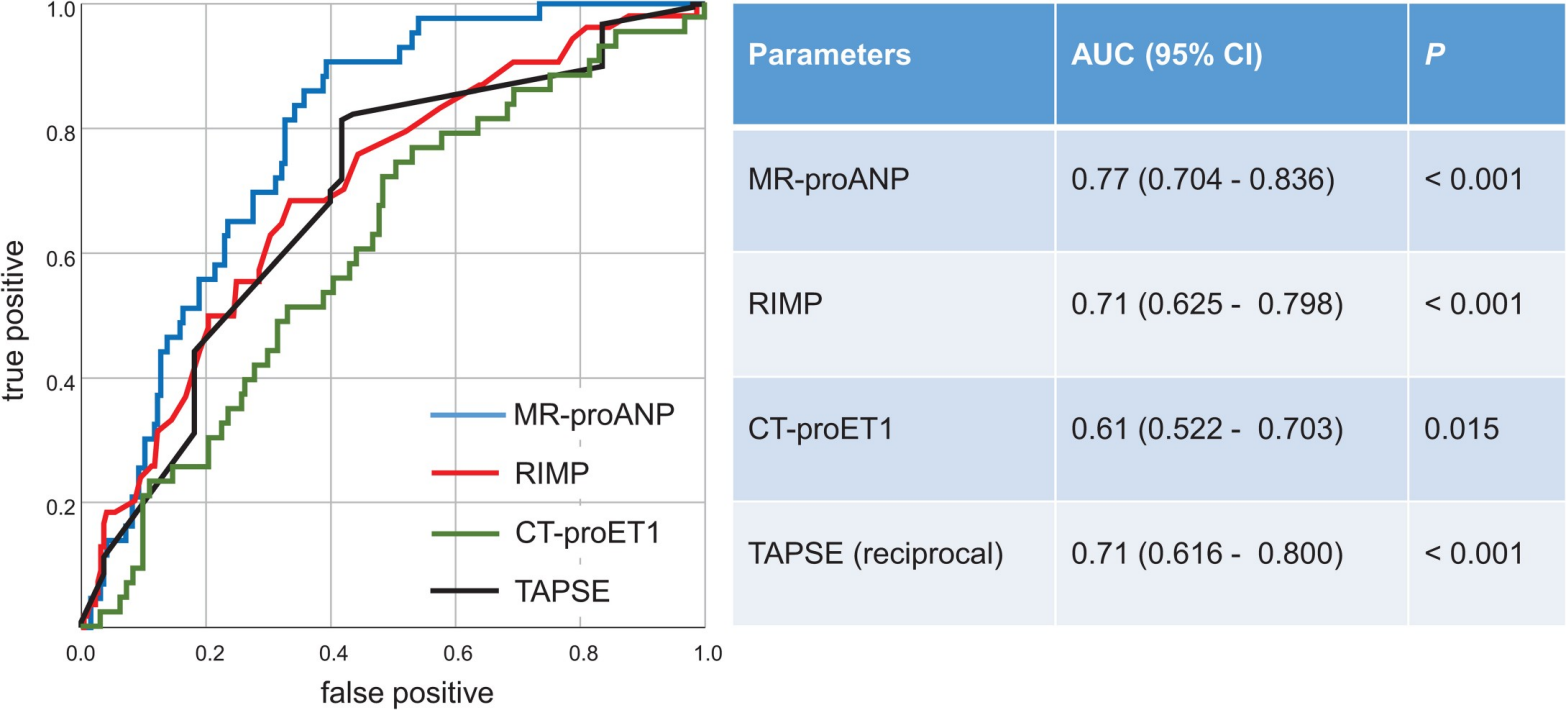
Receiver operating characteristic curves for predicting BPD/death in preterm infants <32 weeks of gestation.

Furthermore, we found that both parameters RIMP and birth weight were also independent predictors of duration of supplemental oxygen (*P* < 0.001 and *P* = 0.048, respectively). Receiver operating characteristic (ROC) curve analysis for the detection of BPD/death revealed that RIMP and TAPSE showed similar areas under the curve (AUC), MR-proANP the largest and CT-proET1 the smallest AUC (Fig 3). With a cutoff value of RIMP determined as 0.24, the diagnostic accuracy was sensitivity 0.70, specificity 0.61, positive predictive value 0.29, and negative predictive value 0.89.

## Discussion

In the present study, we confirmed the hypothesis that the echocardiographic indices RIMP and TAPSE as well as the vasoactive peptides MR-proANP and CT-proET1 on DOL 7 are associated with BPD/death in very preterm infants. RIMP values and vasoactive peptides levels were significantly higher whereas TAPSE values were significantly lower in infants with a subsequent diagnosis of BPD/death compared to controls. However, after correction for co-factors, only RIMP was significantly associated with BPD/death. Early echocardiography seems to be a promising tool for early identification of infants at risk for subsequent BPD development.

To our knowledge, this is the largest study assessing the value of the echocardiographic indices RIMP and TAPSE for prediction of BPD in preterm infants. At 7 days of life, we observed higher RIMP and lower TAPSE values in infants with subsequent BPD development or death. However, the association between TAPSE and BPD/death did not persist after correction for birth weight. This confirms results from our previous study of elevated RIMP values in 121 preterm infants in the first weeks of life with incipient BPD [11]. Seo and Choi measured echocardiographic indices in a study of 67 preterm infants of < 30 weeks GA within the first 14 days (range 2–14 days) of life. They observed higher RIMP and lower TAPSE values in infants with symptomatic early pulmonary hypertension but could not find an association with subsequent BPD development [25]. Sehgal et al. examined the association of right ventricular function with BPD in infants at a much later age of 36 weeks PMA. Similar to our findings, these authors observed elevated RIMP and lower TAPSE values in infants with BPD compared to controls, and an association with the duration of subsequent respiratory support [26].

James et al. studied TAPSE in stable preterm infants < 29 weeks GA in the first 48 hours of life. Although measured in the early transitional period, TAPSE values were in a similar range compared to our study. No associations with clinical outcomes were studied [27]. Our results are also within the range of previously established reference values of preterm infants within 48 hours after birth [28]. Mendez-Abad et al. measured TAPSE in a prospective cohort study at regular intervals from 24 h after birth until 36 weeks PMA and found an association of TAPSE at DOL 14 with the development of BPD in a multivariable model including GA, PMA, N-terminal pro B type natriuretic peptide, lateral triscuspid E’ and A’ waves [13].

We observed higher MR-proANP levels on DOL 7 in infants with subsequent BPD or death compared to infants without BPD. Previous studies on natriuretic peptides and BPD assessed the value of BNP but not MR-proANP [29]. These studies observed an association between BNP levels measured in the first weeks of life and the development of BPD. Similar to elevated MR-proANP levels in the BPD/death group, we observed higher levels of CT-proET1 in this group compared to infants without BPD. Several previous studies have reported associations between CT-proET1 levels and BPD. El Sayed et al. demonstrated that in infants with respiratory distress syndrome, ET-1 was significantly higher in those with subsequent BPD development [30]. In multivariable logistic regression analysis of our results with birth weight, RIMP, TAPSE, MR-proANP and CT-proET1 in the equation, only birth weight and RIMP remained independent predictors of BPD/death. In a previous study of our group including 227 preterm infants, plasma CT-proET1 was measured at birth, DOL 2, 3, 6, and 28 [16]. The predictive value of CT-proET1 for BPD development was highest on DOL 6. In contrast to our current study, Baumann et al. showed a persistent association of CT-proET1 with BPD/death even after correction for clinical factors [16].

There is a growing body of evidence that early assessment of myocardial function and vasoactive peptide levels can indicate subsequent development of BPD. Specifically, early pulmonary hypertension and associated RV strain in the first weeks of life seem to be associated with subsequent BPD development [31,32].

We studied two echocardiographic indices of right ventricular function, RIMP and TAPSE in very preterm infants. Both indices are established markers in adults for echocardiographic right heart assessment but their value for the prediction of BPD is not well known [33]. Mourani et al. performed echocardiographies in 277 preterm infants on day 7 of life. They observed an association between septal wall flattening and right ventricular dilatation with late pulmonary hypertension and BPD [31]. Mirza et al. assessed pulmonary hypertension in 120 preterm infants at 10–14 days of life. They also found markers of early pulmonary hypertension (right ventricular pressure gradient and septal position) to be more common in infants subsequent moderate/severe BPD or death [32]. RIMP is an index of global right ventricular function which depends on pulmonary arterial pressure, myocardial performance and right ventricular afterload [34]. RIMP assesses RV systolic and diastolic function as inflow and outflow measurements of the right ventricle determine RIMP values. When RV pressure increases above that of right atrial pressure, the tricuspid valve closes and marks the end of right ventricular diastole. Increased right ventricular pressures in pulmonary hypertension can therefore influence diastolic filling. The filling of the right ventricle usually consists of passive phase and an active phase driven by right atrial contraction. The ventricular filling in very preterm infants seems to have a greater dependence on atrial contraction [35]. Echocardiographic indices of RV strain might also be dependent on clinical interventions and conditions affecting RV preload or afterload. However, we could not find an association of mechanical ventilation and hsPDA after correction for birth weight and RIMP in our population. We observed an association of TAPSE with BPD/death at 7 days in univariable analysis, but not after adjustment for birth weight. However, Koestenberger et al. have previously shown a strong correlation of TAPSE with GA and birth weight in a cross-sectional study of preterm and term healthy neonates, indicating an association in thoracic and cardiac size which the authors interpreted as a result of maturation and growth [28]. TAPSE could still be a useful marker of incipient BPD but did not prove useful in our study population measured at 7 days of life probably due to the wide range of birth weights of included infants (375–2350 g).

Atrial natriuretic peptide (ANP) is secreted by stressed and distended atrial myocytes in response to volume and pressure load. Since pulmonary hypertension results in increased volume and pressure load, it is biologically plausible that elevated ANP levels are associated with BPD development.

Endothelin-1 is a vasoactive peptide, which is most abundant in the lungs, secreted by endothelium, smooth muscle, airway epithelium, and a variety of other cells [36]. Secretion of Endothelin-1 is stimulated by shear stress, hypoxia, cytokines, growth factors, and lipopolysaccharides. It is elevated in infants and adults with respiratory distress syndrome [36].

Endothelin-1 causes vasoconstriction and is involved in pulmonary vascular disease and fibrosis. Since these features are hallmarks of BPD, Endothelin-1 is likely to play a role as an early marker of BPD development. However, multivariable analysis showed that elevated CT-proET1 levels on DOL 7 were not associated with BPD/death after correction for birth weight, RIMP, TAPSE and MR-proANP. Determination of CT-proET1 levels at different time points or in different population, e.g. focusing on extremely preterm infants might result in different findings.

Strengths of our study include a relatively large sample size and the prospective two-center design. A potential limitation involves the rather low rate of preterm infants with moderate to severe BPD/death in our study (22%) which was lower than previously reported [3]. A potential weakness of our study is that RIMP was measured by pulse-waved Doppler imaging. This requires two echocardiographic samples with near-identical R-R intervals. RIMP estimated from tissue Doppler imaging might be more accurate as it is derived from a single sample.

Prevention of BPD and its long-term sequelae remain elusive due to multifactorial origin and different disease endotypes [37]. Early detection of incipient BPD by plasma biomarkers or echocardiographic indices could promote early identification of at-risk infants who might benefit from timely therapeutic interventions We could show that echocardiographic measurement of RIMP is associated with the development of BPD/death when assessed as early as on DOL 7. A combination of biomarkers with echocardiographic indices did not improve BPD prediction. We think that echocardiographic indices of right ventricular function warrant further validation in future studies.

## Conclusions

Echocardiographic indices, in particular RIMP obtained on DOL 7, seem to be useful for early identification of preterm infants at high risk for BPD or death. This echocardiographic marker might help in the future to identify selected patients who might benefit from early prevention trials.

## Supporting information

S1 Dataset (XLSX)
